# Supplementary material for: The Anatase-to-Rutile Phase Transition in Highly Oriented Nanoparticles Array of Titania with Photocatalytic Response Changes
Source: Nanomaterials (Basel). 2022 Dec 11;12(24):4418. doi: 10.3390/nano12244418 (PMC9785057; doi:10.3390/nano12244418)
Supplement: Supplementary file 1 [file nanomaterials-12-04418-s001.zip › nanomaterials-2065520-supplementary.pdf]

Supplementary Materials to:

The Anatase-To-Rutile Phase Transition in Highly Oriented Nanoparticles Array of Titania with Photocatalytic Response Changes

Olga Boytsova [1,2]*, Irina Zhukova[1], Artem Tatarenko[1], Tatiana Shatalova[2], Artemii Beiltiukov[3], Andrei Eliseev [1], Alexey Sadovnikov [4,5]

1 Department of Materials Science, Lomonosov Moscow State University, Building 73, Leninskie Gory 1, 119991 Moscow, Russia; irinazukova707@gmail.com (I.Z.), tatarenko.artem.h2o@yandex.ru (A.T.), eliseev@inorg.chem.msu.ru (A.E.)
2 Department of Chemistry, Lomonosov Moscow State University, Building 3, Leninskie Gory 1, 119991 Moscow, Russia, shatalovatb@gmail.com (T.S.);
3 Udmurt Federal Research Center of UB RAS, T. Baramzina Str. 34, 426067 Izhevsk, Russia; beltukov.a.n@gmail.com
4 Kurnakov Institute of General and Inorganic Chemistry RAS, Leninskii Prosp. 31, 119071 Moscow, Russia; trinki13@gmail.com (A.S.)
5 Topchiev Institute of Petrochemical Synthesis, Russian Academy of Sciences, Leninskii Prosp. 29, 119991 Moscow, Russia; trinki13@gmail.com (A.S.)
* Correspondence: boytsova@gmail.com(O.B.)

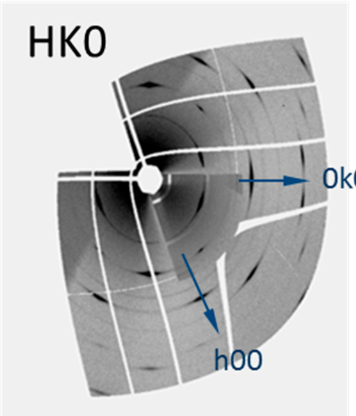

(a)

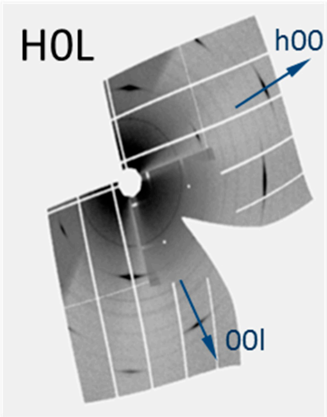

(b)

Figure S1. The HK0 (a) and the H0L (b) reciprocal space layers of an individual crystal of TiO_2 obtained after 2 h at 450 °C, respectively. Data were collected at the Swiss-Norwegian beamline BM01 at the European Synchrotron Radiation Facility (ESRF) in Grenoble, France

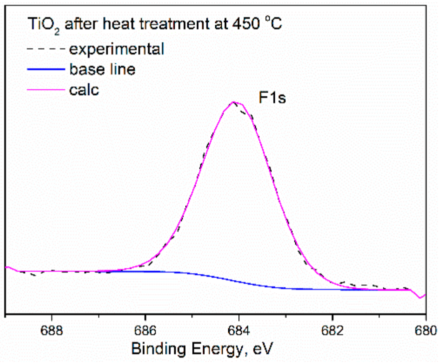

(a)

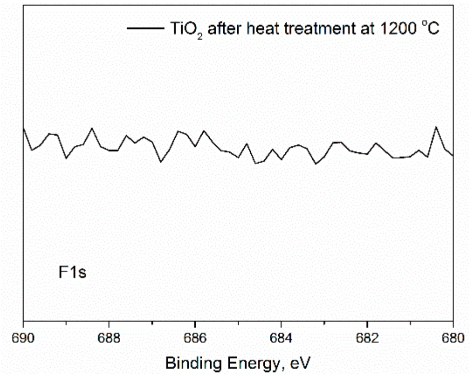

(b)

Figure S2. XPS spectra of F1s for the titanium dioxide after 450 °C (a) and 1200 °C(b)

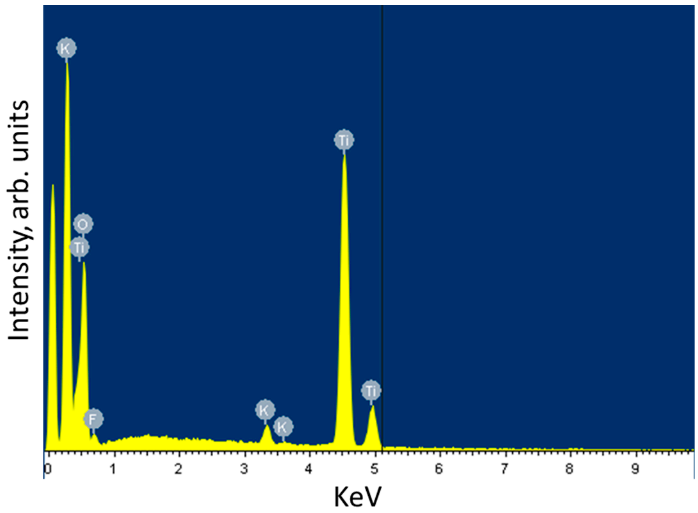

Figure S3. EDX spectra of TiO_2 obtained after annealing of NH_4TiOF_3 (PEG 400) at 1000 °C.

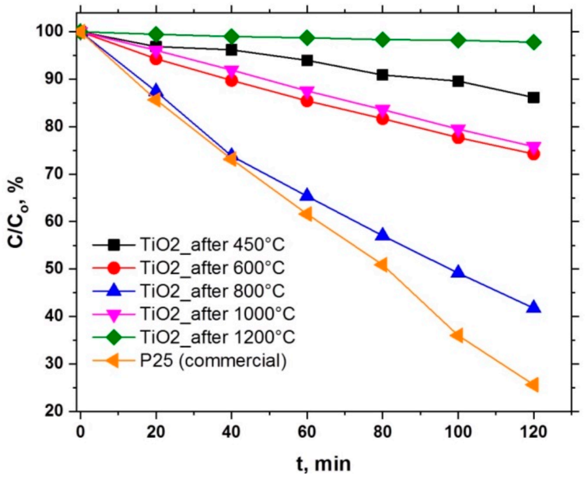

Figure S4. Crystal violet dye photodecomposition rates in the presence of various samples of titanium dioxide (after 450, 600, 800, 1000 and 1200 °C heat treatment and reference - Evonik Aeroxide® TiO_2 P25).
